# Loss of *Ikbkap* Causes Slow, Progressive Retinal Degeneration in a Mouse Model of Familial Dysautonomia

**DOI:** 10.1523/ENEURO.0143-16.2016

**Published:** 2016-09-27

**Authors:** Yumi Ueki, Grisela Ramirez, Ernesto Salcedo, Maureen E. Stabio, Frances Lefcort

**Affiliations:** 1 Department of Cell Biology and Neuroscience, Montana State University, Bozeman, MT 59717; 2 Department of Cell and Developmental Biology, University of Colorado School of Medicine, Aurora, CO 80045

**Keywords:** familial dysautonomia, IKAP/Elp1, Ikbkap, retinal degeneration, retinal ganglion cell

## Abstract

Familial dysautonomia (FD) is an autosomal recessive congenital neuropathy that is caused by a mutation in the gene for inhibitor of kappa B kinase complex-associated protein (*IKBKAP*). Although FD patients suffer from multiple neuropathies, a major debilitation that affects their quality of life is progressive blindness. To determine the requirement for *Ikbkap* in the developing and adult retina, we generated *Ikbkap* conditional knockout (CKO) mice using a *TUBA1a* promoter-Cre (*Tα1-Cre*). In the retina, *Tα1-Cre* expression is detected predominantly in retinal ganglion cells (RGCs). At 6 months, significant loss of RGCs had occurred in the CKO retinas, with the greatest loss in the temporal retina, which is the same spatial phenotype observed in FD, Leber hereditary optic neuropathy, and dominant optic atrophy. Interestingly, the melanopsin-positive RGCs were resistant to degeneration. By 9 months, signs of photoreceptor degeneration were observed, which later progressed to panretinal degeneration, including RGC and photoreceptor loss, optic nerve thinning, Müller glial activation, and disruption of layers. Taking these results together, we conclude that although *Ikbkap* is not required for normal development of RGCs, its loss causes a slow, progressive RGC degeneration most severely in the temporal retina, which is later followed by indirect photoreceptor loss and complete retinal disorganization. This mouse model of FD is not only useful for identifying the mechanisms mediating retinal degeneration, but also provides a model system in which to attempt to test therapeutics that may mitigate the loss of vision in FD patients.

## Significance Statement

Familial dysautonomia (FD) is classified as a hereditary sensory and autonomic neuropathy (Type III). A classic hallmark of the disease is progressive blindness marked by retinal ganglion cell (RGC) loss and optic nerve atrophy. To investigate the consequences of *Ikbkap* loss in the retina, we generated *Ikbkap* conditional knockout mice using *TUBA1a-Cre*. In the retina, *TUBA1a-Cre* is expressed primarily in RGCs, and *Ikbkap* disruption led to slow, progressive RGC degeneration that was subtype and region specific. This was later followed by indirect photoreceptor loss and complete retinal disorganization. Our data demonstrate that this is a powerful model system that faithfully recapitulates the phenotype and progression of FD blindness and can be used to investigate potential therapeutics to treat retinal degeneration in FD.

## Introduction

Familial dysautonomia (FD; also called Riley–Day syndrome) is a fatal autosomal recessive neurodegenerative disorder that is caused by an intronic mutation in *IKBKAP/Elp1*, the gene for inhibitor of kappa B kinase complex-associated protein (IKAP) (Riley et al., 1949; Anderson et al., 2001; Slaugenhaupt et al., 2001). The T-to-C base change in a splice acceptor site results in variable tissue-specific exon skipping and generates an unstable mRNA that undergoes nonsense-mediated decay, leading to a reduction in IKAP/Elp1 protein, particularly in the nervous system (Cuajungco et al., 2003). The function of IKAP/Elp1 is unresolved, although the preponderance of evidence indicates that it functions as a key subunit of the six-subunit Elongator complex, which is essential for transfer RNA (tRNA) modification (Huang et al., 2005; Esberg et al., 2006; Bauer and Hermand, 2012; Xu et al., 2015). Symptoms of FD include decreased pain and temperature sensation, orthostatic hypotension, tachycardia, labile blood pressure, spinal deformities, and gait ataxia, and the majority of FD patients die by age 40 (Riley et al., 1949; Axelrod, 2002; Palma et al., 2014; Dietrich and Dragatsis, 2016; Norcliffe-Kaufmann et al., 2016).

Although FD patients suffer from multiple severe neuropathies, a major quality-of-life issue as they age is progressive blindness. Until recently, the visual loss was thought to be due to sensory and sympathetic-related corneal keratopathy and neovascularization, poor ulceration healing, and lack of tears (Goldberg et al., 1968; Liebman, 1968). However, recent studies report retinal dysfunctions in FD patients, including decreased visual acuity, poor color vision and central visual field loss, temporal optic nerve pallor, and delay in visual evoked potentials (Mendoza-Santiesteban et al., 2012, 2014). Reduction in retinal nerve fiber thickness, which is due to loss of retinal ganglion cells (RGCs), was observed in retinas of FD patients (Mendoza-Santiesteban et al., 2012, 2014). This loss in retinal nerve fiber was more widespread in older patients, suggesting the progressive nature of RGC degeneration.

Various mouse models of FD have been developed to understand the disease mechanisms (Chevrollier et al., 2008; Dietrich et al., 2011, 2012; Hunnicutt et al., 2012; George et al., 2013; Morini et al., 2016). The *Ikbkap* ^-/-^ genotype is embryonic lethal owing to a failure in neurulation and vasculogenesis (Chen et al., 2009), and mice that were generated with the human point mutation showed no phenotype (Hims et al., 2007; Bochner et al., 2013). In contrast, *Ikbkap* conditional knockout (CKO) and hypomorphic mice have provided useful information on the role of IKAP in peripheral nervous system (PNS) development and maintenance (Dietrich et al., 2012; Hunnicutt et al., 2012; George et al., 2013; Jackson et al., 2014; Morini et al., 2016). Our previous study in a PNS model of FD demonstrated that subsets of PNS neurons die by p53- and activated caspase-3–mediated apoptosis in the absence of *Ikbkap* (George et al., 2013).

Although there is great interest in developing treatments to prevent or delay the progressive retinal degeneration to improve FD patients’ quality of life, no study has been published to date that investigates the progression and causes of FD blindness. To this end, we generated a model system in which the consequences of *Ikbkap* loss in the retina could be investigated. We generated *Ikbkap* CKO mice using *TUBA1a* promoter-driven Cre (*Tα1-Cre*), which targets postmitotic neurons (Gloster et al., 1994; Coppola et al., 2004; Coksaygan et al., 2006). Our CKO mice display typical FD patient symptoms, including small stature, kyphosis, and gait disturbances (Chaverra M, George L, Mergy M, Waller H, Kujawa K, Murnion C, Sharples E, Thorne J, Podgajny N, Personius K, Grindeland A, Ueki Y, Eiger S, Cusick C, Babcock M, Carlson G and Lefcort F, unpublished observations). Here, we show that in the retina, the loss of *Ikbkap* in RGCs leads to their slow, progressive degeneration, with the greatest demise in the temporal retina—the same pattern observed in FD patients (Mendoza-Santiesteban et al., 2014). Interestingly, melanopsin-positive intrinsically photosensitive RGCs are resistant to degeneration even with widespread loss of conventional RGCs. In older CKO retinas, optic nerve inflammation, photoreceptor degeneration, Müller glial activation, and disruption of retinal layers are also observed. This is the first study to explore the consequences of *Ikbkap* loss in the retina, and the study reveals that this model will be invaluable for investigating the molecular and cellular mechanisms mediating the demise of retinal neurons, and ultimately for designing therapeutic targets.

## Materials and Methods

### Animals

All mice were housed at the Montana State University, and protocols were approved by the Montana State University Institutional Animal Care and Use Committee. Both male and female mice were used for this study. *Ikbkap* CKO mice were generated by crossing *Tα1-Cre*, which targets postmitotic neurons (Gloster et al., 1994; Coppola et al., 2004; Coksaygan et al., 2006) and *Ikbkap*-floxed mice (International Knockout Mouse Consortium; Chaverra et al., unpublished observations). Mice were used at ages detailed below, and littermate *Cre^-^;Ikbkap^f/f^* mice were used as controls. To determine Cre expression in the retina, *Tα1-Cre* mice were crossed to mTmG reporter mice (stock #007576; Jackson Laboratory, Bar Harbor, ME; Muzumdar et al., 2007). To analyze endogenous expression of *Ikbkap* in the retina, LacZ reporter mice (*Ikbkap:β-gal*) were used (International Knockout Mouse Consortium; George et al., 2013).

### LacZ staining

Eyes were enucleated and fixed (1% formaldehyde, 0.2% glutaraldehyde, and 0.02% NP-40 in PBS) for 30 min at room temperature. The cornea and lens were removed during the first 5 min. Standard LacZ staining was performed at 37°C overnight. Eyes were then cryoprotected at 30% sucrose/PBS and embedded in optimal cutting temperature compound for cryostat sectioning. Sections were analyzed with a light microscope.

### Hematoxylin and eosin staining

Mice were euthanized with CO_2_, and eyes were marked with a green tattoo dye on the temporal surface. Eyes were then enucleated and fixed overnight in PerFix (20% isopropanol, 2% trichloroacetic acid, 4% paraformaldehyde, and 2% zinc chloride), placed in 70% ethanol at room temperature for at least 24 h, and embedded in paraffin for sectioning. Sections (5 µm) were cut through the center of the eye (determined by the presence of the optic disc) and stained with hematoxylin and eosin (H&E). Standard light microscopy was performed to analyze retinal morphology. The number of photoreceptors lying in a single column spanning the outer nuclear layer (ONL) was counted as described previously (Ueki et al., 2008). Counts were made at intervals of 0.25 mm beginning at the optic nerve head (ONH) and toward both the temporal and nasal retinal hemispheres. Statistical analysis was performed using *t*-test. Data are considered significant when *p* < 0.05.

### Immunohistochemistry

Mice and eyes were prepared as above, and eyes were enucleated and fixed in 4% paraformaldehyde for 30 min at room temperature (cornea/lens removed). After a single PBS wash, eyecups were cryoprotected in 30% sucrose overnight at 4°C and embedded in optimal cutting temperature compound (Sakura Finetek, Torrance, CA) and sectioned at 12–14 μm. For immunohistochemistry (IHC), sections were blocked with animal-free blocker (Vector Laboratories, Burlingame, CA) containing 0.5% Triton X-100 for 1 h at room temperature, then primary antibodies were applied and incubated at 4°C overnight. Primary antibodies used were anti–β-galactosidase (Invitrogen, San Diego, CA), anti-GFP (Invitrogen or Abcam, Cambridge, MA), anti-Otx2 (R&D Systems, Minneapolis, MN), anti-AP2α (Developmental Studies Hybridoma Bank, Iowa City, IA), anti-Brn3 (Santa Cruz Biotechnology, Santa Cruz, CA), anti–RNA-binding protein multiple splicing (RBPMS; PhosphoSolutions, Aurora, CO), anti-GFAP (NeuroMab, Davis, CA), anti-Islet1 (Developmental Studies Hybridoma Bank), anti-Sox9 (EMD Millipore, Billerica, MA), anti-Sox2 (Santa Cruz Biotechnology), anti–choline acetyltransferase (EMD Millipore), and anti-PKD2L-1 (EMD Millipore) antibodies. Sections were washed three times with PBS and incubated with secondary antibodies (Invitrogen; Jackson ImmunoResearch, West Grove, PA) and DAPI (Sigma, St. Louis, MO) for 1 h at room temperature. Sections were coverslipped, and confocal microscopy was performed.

### Retinal flat-mount IHC and Brn3^+^ RGC counting

After fixation (as described above), retinas were isolated, and temporal retinas were marked with a small cut. Nonspecific binding was blocked by incubating with animal-free blocker (Vector Laboratories) containing 0.5% Triton X-100 overnight at 4°C, and anti-Brn3 antibody (Santa Cruz Biotechnology) was applied for 2 days at 4°C. Retinas were washed three times with PBS, incubated with secondary antibodies (Invitrogen) and DAPI (Sigma) overnight at 4°C, washed three times with PBS, and mounted on slides.

### Confocal imaging

Confocal microscopy was performed using a Leica TCS SP8. To ensure quantitative image quality, laser power, pinhole settings, photomultiplier tube settings, and intensity thresholds were kept constant for a given antibody. For quantifying *Tα1-Cre* expression in RGCs, the number of RBPMS^+^, GFP^+^, and RBPMS^+^GFP^+^ cells were quantified at <0.25 mm (central) and 1 mm from the ONH at temporal and nasal hemispheres. For Brn3^+^ RGC counts on flat mounts, confocal images were taken at 1 mm from the ONH in temporal, nasal, superior, and inferior retinas, and the number of Brn3^+^ cells in each image was counted manually. The number of cells in 1 mm^2^ of the retina was calculated and plotted as a percentage of 1-month-old control retinas. Statistical analysis was performed using *t*-test. Data are considered significant when *p* < 0.05.

### Optic nerve analysis

The optic nerve was carefully cut away from the eyecup after 4% paraformaldehyde fix described above, rinsed in PBS (3 × 15 min), and stored in PBS for later use. Nerves were cryoprotected overnight with 20% sucrose in phosphate buffer (PB) and sectioned by cryostat (longitudinally) at 16 μm onto Tanner positively charged slides (Light Labs, Aurora, CO). Slides were then incubated overnight in anti-Iba1 antibody (Wako Chemicals USA, Richmond, VA). The next day, slides were rinsed in 0.1 m PBS and incubated for 2 h with a secondary antibody. The slides were washed two times for 10 min each in PBS and then once for 10 min in PB before being coverslipped with Fluoromount G (Southern Biotech). Optic nerve sections were imaged with 0.5-µm *z*-step sizes using a Leica TCS SP5 Confocal Laser Scanning Microscope. The confocal image stacks were then analyzed using our in-house *imstack* toolbox developed using the MATLAB programming language (Mathworks, Natick, MA). Briefly, we loaded each *z*-stack into the *imstack* toolbox. We then drew a rectangular region of interest (ROI) with a maximum area contained entirely within the optic nerve and extended the ROI throughout the *z*-stack, ensuring that the 3D ROI did not capture any area outside of the optic nerve. This 3D ROI established our analysis volume. We next calculated a threshold value using the method of Otsu (1979) and adjusted this value to accommodate signal-to-noise ratios across the entire experimental image dataset. This threshold value was used to threshold pixels inside the ROI. Pixels with intensity values above the adjusted threshold value were designated as Iba1^+^ signal. All positive pixels were counted and then divided by the total number of pixels contained within the analysis volume to establish the Iba-positive ratio. For each optic nerve, at least two separate 3D ROIs were established and analyzed, and these results were averaged. For final comparison between the mutant and the control, we averaged our results across optic nerves from within a group and used a two-tailed *t*-test to determine significance between groups.

### RGC subtype analysis

Six- and 9-month control and mutant retinas were fixed as described above, and flat-mount IHC was performed to count RGCs. Retinas were washed in PBS (3 × 15 min) and incubated for 3 h in blocking solution (5% donkey serum, 1% bovine serum albumin, and 0.5% Triton X-100 in 0.1 m PBS). Retinas were incubated in anti-RBPMS (PhosphoSolutions), anti-melanopsin (Advance Targeting Systems, San Diego, CA), and anti–OPN1-SW (Santa Cruz Biotechnology) antibodies for 2 d at 4°C, washed (6 × 10 min), and incubated in secondary antibodies for 3 h at room temperature. Retinas were washed in PBS (3 × 15 min) followed by PB (1 × 15 min). They were mounted ganglion cell layer (GCL) up on glass slides with Fluoromount-G (Southern Biotechnology, Birmingham, AL) with special attention to the superior and inferior orientation of the retina. The orientation of the retina (dorsal/superior versus ventral/inferior leaflets) was confirmed by immunostaining with s-opsin, which is expressed at higher concentration in the ventral (inferior) retina (Applebury et al., 2000). Whole-mount retinas were imaged using a Leica TCS SP5 Confocal Laser Scanning Microscope. For retinas, a tile scan of the entire retina at the outer retinal layers was acquired at 10× to orient the retina based on the s-opsin gradient. Then, *z*-stacks at 1.5-µm increments and *xy*-dimension of 0.15 mm^2^ were acquired through the RGC layer at 40×. Two adjacent but nonoverlapping stacks were acquired 1 mm from the ONH in each of the four quadrants of the retina (superior, nasal, inferior, and temporal). Bleed-through was avoided by using a sequential scan setting. Cells were manually counted in Adobe Photoshop CC 2015; only RGCs in which the entire soma profile was shown within the image screen were counted. Only melanopsin ganglion cells with bright staining in which proximal portions of the primary dendrites were visible were counted. Data analysis and statistics were performed using appropriate one- or two-way ANOVA. Tukey’s honestly significant difference test was used to assess statistically significant main effects or interactions. Data are considered significant when *p* < 0.05.

## Results

### *Ikbkap* is expressed in many types of postmitotic neurons of the retina

Because patients with FD suffer from blindness, which generally starts in their twenties (Mendoza-Santiesteban et al., 2012, 2014), there is considerable interest in developing treatments to ameliorate blindness and prevent progressive degeneration. The purpose of this study was to generate a model system in which we could identify the retinal cell types affected and ultimately determine the mechanisms causing blindness in FD. Generation of an FD blindness model would also prove powerful for testing potential treatments. Toward this goal, it is critical that we understand the functions and retinal cell types in which *Ikbkap* is required. Although there is clinical evidence for retinal degeneration in FD patients (Mendoza-Santiesteban et al., 2014), the expression of *Ikbkap* and its requirement in the retina have not been investigated.

**Figure 1.**
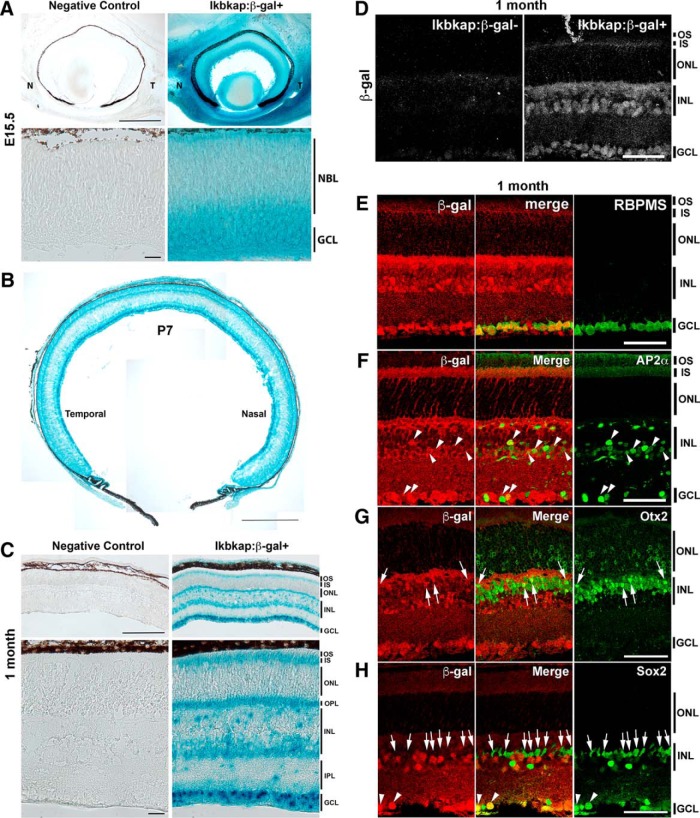
Endogenous *Ikbkap* expression in the retina. Representative LacZ staining on *Ikbkap:β-gal* retina or β-galactosidase IHC images are shown. ***A***, At E15.5, *Ikbkap* expression was detected in developing RGCs in the GCL. N, nasal; T, temporal. ***B***, P7 retina showed strong expression in the GCL. There was no regional bias or asymmetry in the expression pattern. ***C***, At 1 month, RGCs, amacrine cells, subset of bipolar cells, and photoreceptors (IS and outer plexiform layer) expressed IKAP. ***D***, At 1 month, antibody staining showed the same pattern of *Ikbkap* expression as LacZ staining. ***E***, All RGCs (RBPMS^+^, green) expressed *Ikbkap* (red; bottom). ***F***, Many amacrine cells (AP2α^+^) expressed *Ikbkap* (red; arrowheads). ***G***, A subset of bipolar cells (Otx2^+^ in INL) expressed *Ikbkap* (red; arrows). ***H***, Müller glial marker Sox2 (green) did not colocalize with β-gal (red; arrows), suggesting that they do not express *Ikbkap*. Some of the Sox2^+^ amacrine cells expressed *Ikbkap* (arrowheads). NBL, neuroblastic layer. Scale bars, 1 mm (***A***, top), 50 μm (***A***, bottom), 500 μm (***B***), 250 μm (***C***, top), 25 μm (***C***, bottom), and 50 μm (***D–H***).

Toward this end, we first determined the expression pattern of *Ikbkap* in wild-type retinas at various developmental ages using LacZ reporter mice (*Ikbkap:β-gal*; George et al., 2013). Retinas at embryonic day 15 (E15; peak of RGC generation), postnatal day 7 (P7), P14 (completion of retinal development), 1 month, and 2 months (adult) were collected, and LacZ staining was performed (Fig. 1*A–C*). At E15, *Ikbkap* expression was detected in postmitotic neurons in the GCL but not in retinal progenitors (Fig. 1*A*). We did not observe any regional bias or asymmetry in the expression pattern at any developmental stage (Fig. 1*A*, *B*). At P14 and older, the mature expression pattern was established: LacZ staining was detected in RGCs, in a subset of cells in the inner nuclear layer (INL), photoreceptor inner segment (IS), and outer plexiform layer (Fig. 1*C*, 1 month shown). IHC analyses using anti–β-galactosidase antibody at 1 month showed the same pattern as the LacZ staining (Fig. 1*D* top) and revealed that all RGCs express *Ikbkap*, indicated by the colocalization of β-galactosidase and a pan-RGC marker, RBPMS (Rodriguez et al., 2014; Fig. 1*E*). In addition, *Ikbkap* expression was detected in many amacrine cells in both the INL and GCL (AP2α^+^ or Sox2^+^; Fig. 1*F*, *H*, arrowheads) as well as in a subset of bipolar cells (Otx2^+^ in INL; Fig. 1*G*, arrows). Interestingly, Sox2^+^ Müller glial nuclei did not colocalize with β-galactosidase (Fig. 1*H*, arrows), suggesting that Müller glia do not express *Ikbkap* in the adult retina.

### Generation of *Ikbkap* CKO mice that model FD optic neuropathy

**Figure 2.**
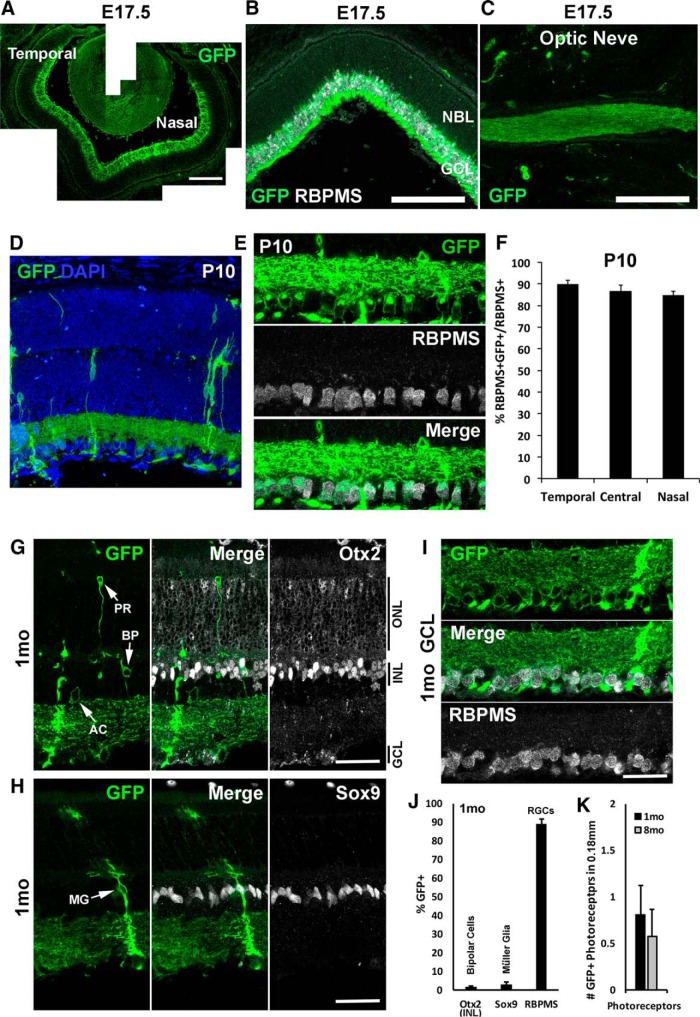
*Tα1-Cre* mice were crossed to mTmG Cre reporter, and GFP expression was analyzed at E17.5, P10, and 1 and 8 months. ***A*** and ***B***, At E17.5, Cre expression (GFP^+^) was detected in the GCL without regional bias. GFP colocalized with pan-RGC marker RBPMS (white). ***C***, GFP was detected in the optic nerve at E17.5, indicating Cre expression in RGCs. ***D***, Very few cells other than RGCs expressed Cre (GFP^+^) at P10. ***E***, Most RGCs (RBPMS^+^) expressed Cre (GFP^+^) at P10. Representative images at the temporal retina are shown. ***F***, Approximately 90% of RGCs expressed Cre by P10, and there was no regional bias. Error bars represent SEM (*n* = 4). Central, >0.25 mm from ONH; temporal and nasal, 1 mm from ONH. ***G–K***, Cell type–specific markers were used to analyze Cre expression in the retina at 1 month. Very few bipolar cells (BP; Otx2^+^ in INL; ***G***, ***J***), amacrine cells (AC; ***G***), photoreceptors (PR; ***G***, ***K***), or Müller glia (MG; Sox9^+^; ***H***, ***J***) expressed Cre, whereas Cre expression was detected in ∼90% of RGCs (RBPMS^+^; ***I***, ***J***). Cre-expressing photoreceptor numbers did not increase in older retinas (8 months) compared with 1 month (***K***). Error bars in ***J*** and ***K*** represent SEM (*n* = 3). NBL, neuroblastic layer. Scale bars, 250 μm (***A–C***) and 50 μm (***G–I***).

We generated *Ikbkap* CKO mice using *Tα1-Cre*, which targets postmitotic neurons (Chaverra et al., unpublished observations; Gloster et al., 1994; Coppola et al., 2004; Coksaygan et al., 2006). To measure Cre expression pattern in the retina, we crossed *Tα1-Cre* with *mTmG Cre* reporter mice (Muzumdar et al., 2007). At E17.5, *Cre* expression was detected in postmitotic cells in the GCL and optic nerve, but not in retinal progenitors (Fig. 2*A–C*). To our surprise, at P10, *Cre* expression was fairly limited to RGCs, with only a few other retinal neuron types and Müller glia being Cre*^+^* (Fig. 2*D*). In the central (>0.25 mm from the ONH), temporal, and nasal (1 mm from the ONH) retina, ∼90% of RGCs (RBPMS^+^) expressed Cre by P10 without regional bias (Fig. 2*E*, *F*). The Cre reporter expression pattern did not change in 1- and 8-month retinas, and there was no increase in Cre^+^ cells compared to P10 retinas (Fig. 2*G–K*; 1 month images shown). Very few bipolar cells (1.6% Otx2^+^ cells in INL; Fig. 2*G*, *J*), photoreceptors (Fig. 2*G*, *K*), Müller glia (3.0% Sox9^+^ cells; Fig. 2*H*, *J*), or amacrine cells (Fig. 2*G*) expressed the GFP Cre reporter, whereas 89.2% of RGCs (Fig. 2*I*, *J*) expressed Cre at 1 month. At 8 months, the number of GFP^+^ photoreceptors did not differ significantly compared to younger (1 month) retinas (Fig. 2*K*).

### Loss of *Ikbkap* causes severe degeneration in older CKO retinas

**Figure 3.**
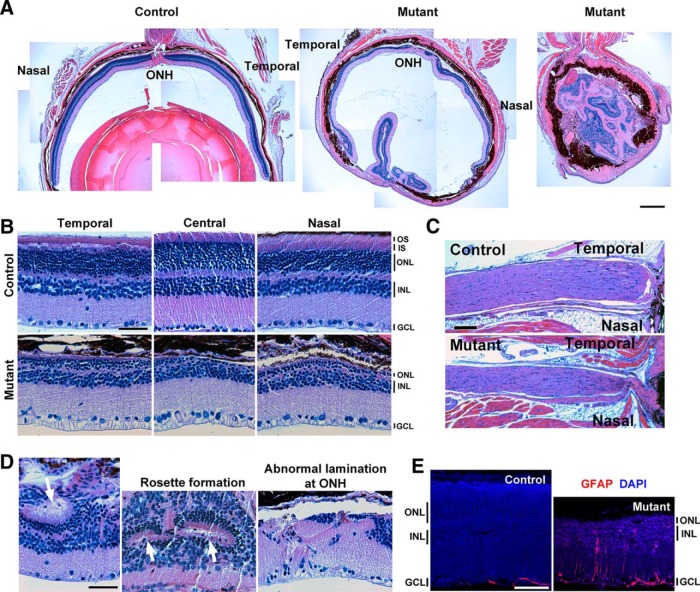
Nineteen-month *Ikbkap* CKOs displayed severe retinal degeneration. ***A***, H&E staining showing a control and two mutant (CKO) eyes. Mutant eyes were smaller, and lenses were often absent; retinas showed obvious sign of degeneration. ***B***, Retinal cross sections at temporal, central, and nasal retinas. Mutant retinas showed clear photoreceptor degeneration, indicated by the thinner ONL and the absence of IS and OS. The number of cells in GCL was reduced. ***C***, CKO optic nerves were thinner. ***D***, Abnormal retinal structures, such as rosette formation and dyslamination of the layers, were observed in the mutant retinas. ***E***, IHC of the GFAP indicated Müller glial activation in the mutant retinas. Scale bars, 250 μm (***A***), 50 μm (***B***, ***D***, and ***E***), and 100 μm (***C***).

Because *Tα1-Cre* is expressed in many postmitotic neurons throughout the central and peripheral nervous system, *Ikbkap* CKO mice die on average by 6 months of age owing to progressive central and peripheral neuropathy as observed in FD patients (Chaverra et al., unpublished observations). However, the phenotype is highly variable between CKOs, and some occasionally survive longer, which allowed us to analyze older CKO retinas. We assessed the overall retinal morphology of 19-month-old CKO retinas and optic nerves by H&E staining (Fig. 3). In general, mutant eyes were smaller, retinal layers were disrupted, and the lenses were often absent (Fig. 3*A*). Many eyes older than 14 months showed immune cells and debris in the vitreous (data not shown). Cross sections of 19-month-old mutant retinas showed obvious photoreceptor degeneration across the retina, with a thinner ONL and an absence of photoreceptor IS and outer segment (OS; Fig. 3*B*). Optic nerves of 19-month-old mutants were clearly thinner than those of their control littermates, an indication of RGC degeneration (Fig. 3*C*). In addition, mutant retina displayed rosette formation and dyslamination of retinal layers (Fig. 3*D*), as well as Müller glial activation (Fig. 3*E*), the hallmarks of retinal degeneration and stress. Whether all or some of these phenotypes were direct or indirect consequences of the loss of *Ikbkap* in RGCs has yet to be determined.

### RGCs are the first cell type to be affected in *Ikbkap* CKO retinas

**Figure 4.**
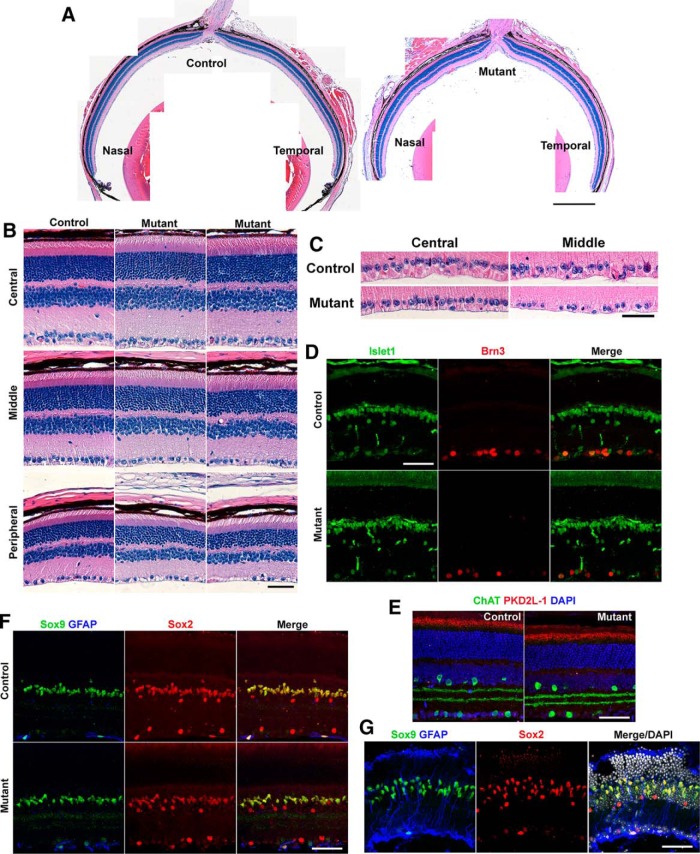
Six-month *Ikbkap* CKO retinas were grossly normal except for the reduction in RGCs. ***A***, H&E staining showing control and mutant (CKO) eyes. The mutant eyes at 6 months appear normal. ***B***, Retinal cross sections at central (>0.25 mm from ONH), middle (1 mm from ONH), and peripheral (1.75 mm from ONH) retinas in the temporal hemisphere. Retinal structure of the mutant was grossly normal, except for the reduction in cell number in the GCL that is apparent toward the peripheral retinas. ***C***, Reduction in cell number was observed in the mutant GCL at 1 mm from the ONH (middle). Images of the temporal retina are shown. ***D***, Mutant retinas showed reduced numbers of RGC marker, Brn3 (red). Islet1 (green) IHC showed normal cholinergic amacrine and optic nerve bipolar cells. ***E***, Choline acetyltransferase (green) IHC showed normal number and structure of cholinergic amacrine cells. PKD2L-1 (red) IHC indicated normal photoreceptor cilia structure. ***F***, Müller glial marker Sox9 (green), Sox2 (red), and GFAP (blue) showed normal, nonactivated Müller glia in the mutant retinas. ***G***, Because of variability in the phenotype, degenerating mutant retinas were occasionally observed at 6 months. In these retinas, Müller glia were activated (GFAP upregulation), and photoreceptor rosettes were seen. ***D–G***, Images represent 1 mm from the ONH at the temporal retina. Scale bars, 250 μm (***A***) and 50 μm (***B–G***).

We analyzed 6-month-old mutant retinas for any sign of retinal degeneration. Although mutant eyes were occasionally smaller compared with their littermate controls, overall morphology of the eye appeared grossly normal (Fig. 4*A*). Analysis of 6-month retinal cross sections by H&E staining showed no abnormal lamination or thickness of retinal layers in the mutants (Fig. 4*B*). However, loss of cells in the middle (1 mm from the ONH) to peripheral retinas was observed in the RGC layer of the mutant retina (Fig. 4*B*, *C*). IHC analyses revealed reduction in Brn3^+^ RGCs in the mutants (Fig. 4*D*, red; see below for quantitation), whereas staining for Islet1 (a marker for cholinergic amacrine, optic nerve bipolar, and RGCs; Fig. 4*D*, green) and choline acetyltransferase (a marker for cholinergic amacrine cells; Fig. 4*E*, green) showed comparable cell numbers, morphology, and location between the mutant and littermate controls. Sox9^+^/Sox2^+^ Müller glial nuclei were localized in a single layer in the INL, and the activation marker GFAP was not observed in either mutant or control retinas (Fig. 4*F*). The number of photoreceptor nuclei appeared normal (Fig. 4*B*), and the photoreceptor cilia marker PKD2L-1 showed a normal pattern in the mutant (Fig. 4*F*). Because of the high variability in mutant phenotypes, we occasionally observed severe retinal degeneration as early as 6 months (Fig. 4*G*), and those retinas had photoreceptor rosette formation, Müller glial activation (GFAP upregulation), and loss of RGCs, as seen in older mutant retinas (Fig. 3).

### Loss of *Ikbkap* in RGCs causes slow, progressive RGC degeneration and optic nerve inflammation

**Figure 5.**
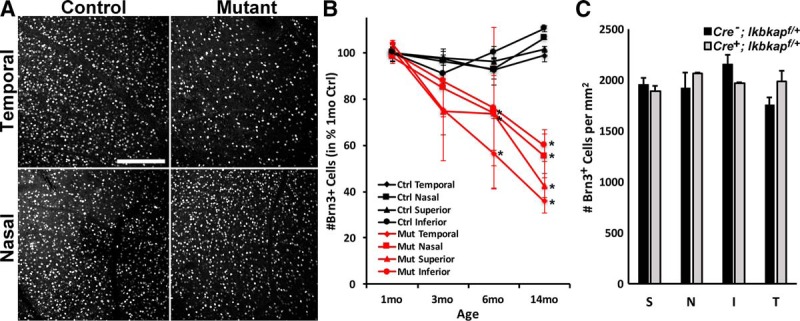
Loss of *Ikbkap* in RGCs caused slow, progressive RGC degeneration. ***A***, Representative Brn3 (RGC marker) staining in the temporal and nasal retinas at 6 months. Images were taken at 1 mm from ONH. ***B***, The number of Brn3^+^ RGCs in *Ikbkap* CKO (mutant) retinas was counted. Significant loss of Brn3^+^ cells was observed in temporal and superior retinas at 6 months, which progressively spread into entire retinas by 14 months. ***C***, The numbers of Brn3^+^ RGCs were counted in each quadrant of 6- to 8-month-old *Cre^-^;Ikbkap^f/+^* and *Cre^+^;Ikbkap^f/+^* retinas at 1 mm from the ONH. There was no significant decrease in the number of RGCs in *Cre^+^;Ikbkap^f/+^* compared to *Cre^-^;Ikbkap^f/+^*retinas, demonstrating that Cre expression itself and/or loss of one *Ikbkap* allele did not cause RGC degeneration. S, superior; N, nasal; I, inferior; T, temporal. Error bars represent SEM (*n* = ≥4 per point for ***B***; *n* = 3 for ***C***). *p < 0.05 with *t*-test. Scale bars, 100 μm (***A***).

Because *Tα1-Cre* is expressed predominantly in RGCs in the retina (Fig. 2) and human FD patients show RGC defects (Mendoza-Santiesteban et al., 2012, 2014), we tracked spatial and temporal changes in RGCs in the *Tα1-Cre Ikbkap* CKO retinas. Retinas were harvested at 1, 3, 6, and 14 months of age, and the number of RGCs 1 mm from the ONH in temporal, nasal, superior, and inferior retinas were counted using the RGC nuclear marker Brn3 (Fig. 5). At 1 month, the mutant and control retinas contained similar numbers of Brn3^+^ RGCs in all quadrants, suggesting that RGC development was not affected by the absence of *Ikbkap*. In contrast, by 6 months, a significant loss of RGCs was observed in the mutant retinas. The reduction in RGCs was greatest in the temporal retina (∼50% reduction; Fig. 5*A*, *B*), which corresponds to the clinical observation of FD patients (Mendoza-Santiesteban et al., 2012, 2014). Superior and inferior mutant retinas had an approximately 30% reduction in RGCs compared with control retinas at 6 months. RGC degeneration continued with age, and by 14 months, panretinal loss of RGCs was observed, with the most dramatic loss of RGCs, greater than 60%, in the temporal retina. To rule out a possibility that Cre expression itself causes RGC degeneration, the number of Brn3^+^ RGCs in 6- to 8-month *Cre^-^;Ikbkap^f/+^* and *Cre^+^;Ikbkap^f/+^* retinas was counted at 1 mm from the ONH (Fig. 5*C*). The results showed there was no loss of RGCs, indicating that neither Cre expression itself nor loss of one *Ikbkap* allele causes RGC degeneration. We conclude from these results that in the absence of *Ikbkap*, RGCs undergo a slow but progressive degeneration, with the same spatial pattern as observed in FD patients.

**Figure 6.**
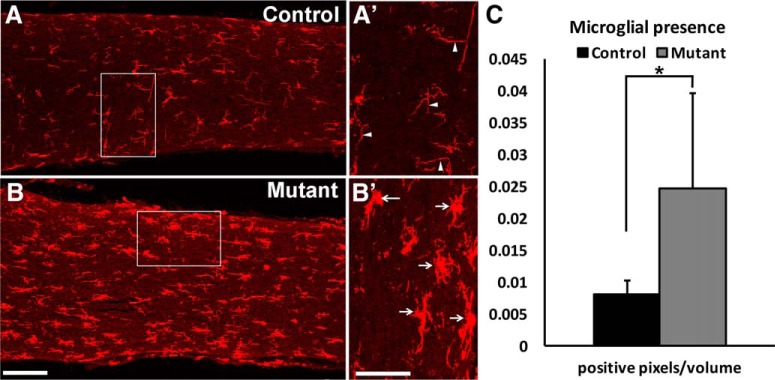
Activation of microglia was observed in the *Ikbkap* CKO optic nerves. Maximum-intensity *z*-stack projections of longitudinal optic nerve sections were stained with microglial/macrophage marker Iba1 in 9-month control (***A***) and mutant (***B***) optic nerves. Representative images are shown. Microglia in control optic nerves had thin ramified branches (arrowheads in ***Aʹ*** inset); microglia in mutant optic nerves had ameboid morphology (arrows in ***Bʹ***) indicative of inflammatory response. White boxes in ***A*** and ***B*** indicate areas imaged at higher magnification in ***Aʹ*** and ***Bʹ***, respectively. (***C***) The number of Iba1-positive pixels that met the criteria was counted and divided by the total number of pixels in the volume of the area, as described in Materials and Methods. The data show that mutant microglia occupied increased areas in the optic nerve, suggesting the presence of inflammatory response. *p = 0.05 with a two-tailed *t*-test (*n* = 6 for control and *n* = 5 for mutant). Scale bars, 100 μm (***A*** and ***B***) and 50 μm (***Aʹ*** and ***Bʹ***).

We also analyzed the optic nerves (RGC axon bundle) of 6- and 9-month mutant and control mice. Although the circumference of the optic nerves was slightly smaller in both 6- and 9-month mutant optic nerves compared with controls, the difference was not significant (data not shown). A microglial/macrophage marker, Iba1 (Ito et al., 2001), showed no difference in microglial number and morphology in the 6-month mutant and control optic nerves (data not shown). However, by 9 months, the optic nerves of mutant animals demonstrated evidence of inflammation (Fig. 6; Ito et al., 2001). Whereas control optic nerves showed microglia with thin ramified branches, mutant optic nerves had microglia with ameboid morphology (Fig. 6*A*, *B*). Our analysis shows increased infiltration of activated microglia in the optic nerves of mutant mice (Fig. 6*C*).

### Melanopsin^+^ RGCs are resistant to degeneration caused by the absence of *Ikbkap*

**Figure 7.**
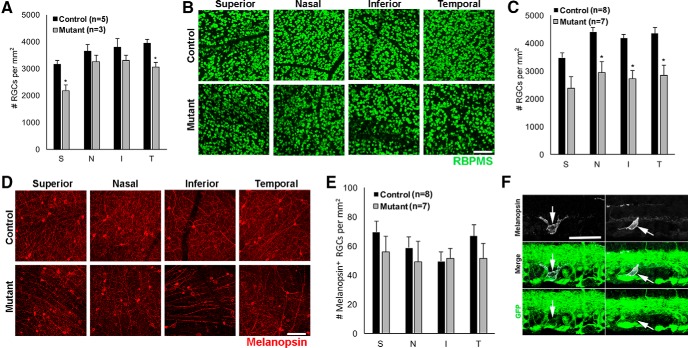
Melanopsin^+^ ipRGCs were resistant to degeneration. All images were taken at 1 mm from the ONH, and numbers of RBPMS^+^ (total RGC marker) or melanopsin^+^ (ipRGC marker) cells were counted manually in superior (S) nasal (N), inferior (I), and temporal (T) leaflets. ***A***, At 6 months, the mean number of RGCs was significantly lower for mutant mice in superior and temporal retinas (*p* = 0.01 and 0.02 with ANOVA and Tukey’s honestly significant difference test). ***B***, Representative RBPMS labeling of the 9-month control and mutant retinas. ***C***, At 9 months, the mean number of RGCs was significantly lower for mutant mice in nasal, inferior, and temporal retinas (*p = 0.02, 0.03, and 0.02 with ANOVA and Tukey’s honestly significant difference test). ***D***, Representative melanopsin labeling of 9-month control and mutant retinas. ***E***, At 9 months, there was no significant difference in ipRGC counts between control and mutant retinas. ***F***, Cre expression in melanopsin^+^ RGCs was analyzed in *Tα1-Cre;mTmG* Cre reporter retinas at P10. Ten melanopsin^+^ cells were found in three different Cre reporter retinas, and all 10 were GFP^+^, suggesting that ipRGCs express Cre. Two representative cells are shown (arrows). Error bars in ***A***, ***C***, and ***E*** represent SEM. Scale bars, 100 μm (***B*** and ***D***) and 50 μm (***F***).

Although many mature mouse RGCs (80%–85%) are known to be Brn3^+^ (Brn3a, Brn3b, and/or Brn3c) and Brn3 is an established nuclear marker for RGCs (Xiang et al., 1995; Nadal-Nicolas et al., 2009; Galindo-Romero et al., 2011), we also quantified the number of RBPMS^+^ RGCs, since the RBPMS antibody selectively and exclusively labels all RGCs (Rodriguez et al., 2014). We immunostained whole-mounted retinas at 6 and 9 months of age and counted RBPMS^+^ cells 1 mm from the optic nerve disk in the superior, nasal, inferior, and temporal retina. In 6-month mutant retinas, we observed a 20%–30% reduction in RGCs in the superior and temporal retina (*p* = 0.01 and 0.02, respectively; Fig. 7*A*). There was temporal-superior bias in total (RBPMS^+^) RGC loss, similar to Brn3^+^ RGC degeneration (Fig. 5). In 9-month mutant retinas, more widespread reduction (30%–35% decrease) in RGCs was observed; reduction in nasal, inferior, and temporal retinas were significant (*p* = 0.02, 0.03, and 0.02, respectively; Fig. 7*B*, *C*). RGC counts were highly variable between mutants at the same age, possibly because of variability in Cre penetrance. We also immunostained using antimelanopsin antibody (Fig. 7*D*), which is known to label M1–M3 subtypes of intrinsically photosensitive RGCs (ipRGCs). ipRGCs express the photopigment melanopsin and are photosensitive to light independently of rods and cones (Berson et al., 2002; Estevez et al., 2012). Interestingly, we did not observe a reduction in melanopsin^+^ RGC in either 6- or 9-month mutant retinas (Fig. 7*E*; 6-month data not shown). Cre reporter analysis showed that melanopsin^+^ ipRGCs were GFP^+^ at P10 (Fig. 7*F*), indicating Cre expression in this RGC subtype. In addition, our endogenous *Ikbkap* expression analysis of the retina at 1 month also showed that all the RGCs (RBPMS^+^) expressed *Ikbkap* (β-gal^+^; Fig. 1*E*). These results suggest that melanopsin^+^ RGCs are resistant to degeneration in the absence of *Ikbkap*.

### RGC loss is followed by photoreceptor degeneration in *Ikbkap* CKO retinas

**Figure 8.**
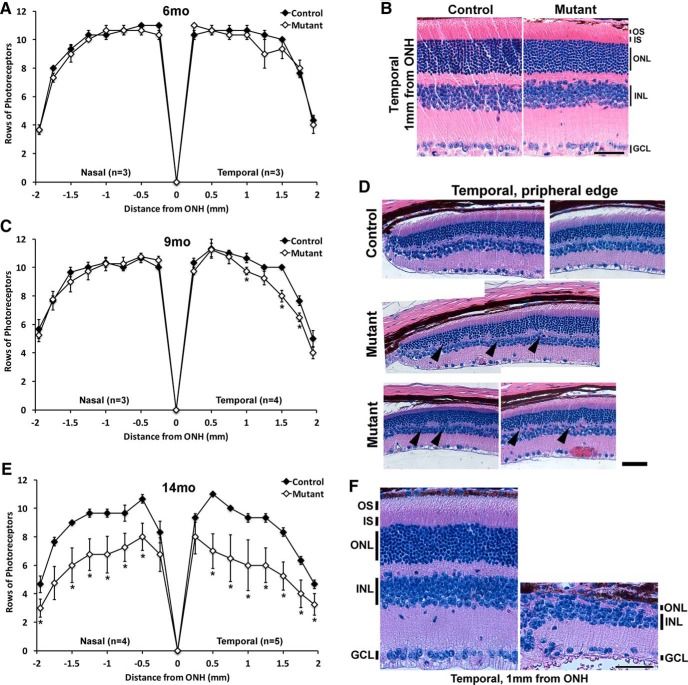
Photoreceptor degeneration was observed in *Ikbkap* CKO retinas. The number of rows of photoreceptor nuclei in the ONL was counted at 0.25 mm from the optic nerve in the temporal and nasal retinas at 6 months (***A***), 9 months (***C***), and 14 months (***E***). H&E-stained cross sections. ***A*** and ***B***, At 6 months, no sign of photoreceptor degeneration was observed. ***C*** and ***D***, At 9 months, significant loss of photoreceptors and disorganization of ONL (arrowheads) in the mutant temporal and peripheral retinas were observed. ***E*** and ***F***, At 14 months, the number of rows of photoreceptor nuclei was clearly reduced across the retina. In addition, photoreceptor IS and OS were absent. Error bars in ***A***, ***C***, and ***E*** represent SEM (*p < 0.05 with *t*-test). Scale bars, 50 μm (***B***, ***D***, and ***F***).

Because older mutant retinas show clear signs of photoreceptor degeneration (Fig. 3), we quantified the number of photoreceptors in 6-, 9-, and 14-month mutant and control retinas (Fig. 8). The number of photoreceptors lying in a single column spanning the ONL was counted in H&E-stained retinal cross sections as described previously (LaVail et al., 1992; Ueki et al., 2008; Chollangi et al., 2009). Counts were made at 0.25-mm intervals beginning at the ONH and toward both the temporal and nasal retinal hemispheres (Fig. 8*A*, *C*, *E*). At 6 months, mutant retinas had similar numbers of rows of photoreceptor nuclei in the ONL across the retina, and photoreceptor IS and OS showed normal morphology (Fig. 8*A*, *B*). By 9 months, the temporal retina of the mutants showed slight but significant reduction in photoreceptor nuclei, indicative of photoreceptor degeneration (Fig. 8*C*). We also observed dyslamination of ONL at temporal peripheral (>1.5 mm from ONH) retinas of mutants, with the ectopic presence of photoreceptor nuclei in the outer plexiform layer (Fig. 8*D*, arrowheads). By 14 months, panretinal photoreceptor degeneration was observed: on average, the number of rows of photoreceptor nuclei was reduced by 30%–40% across mutant retinas compared with controls (Fig. 8*E*). The extent of photoreceptor loss varied between mutants, and some mutant retinas displayed severe photoreceptor degeneration with no IS and OS (Fig. 8*F*). We speculate that this photoreceptor degeneration in our *Ikbkap* CKO retinas is an indirect consequence of *Ikbkap* loss in cell types other than photoreceptors, since *Tα1-Cre* expression is nearly absent in photoreceptors in both young and older retinas (Fig. 2*A*, *K*).

## Discussion

Familial dysautonomia is an autosomal recessive congenital neuropathy caused by an intronic mutation in *IKBKAP*, which encodes the protein IKAP (also known as ELP1). Although one of the major adversities that affects FD patient quality of life is progressive blindness, and there is great interest in developing treatments to ameliorate and prevent progressive blindness in the FD community, development of model systems and the pathophysiological mechanisms underlying the loss of vision have not been the focus of any study. We developed and characterized the retinal phenotype of a new mouse model of FD, in which *Ikbkap* is disrupted in postmitotic neurons (*Tα1-Cre Ikbkap* CKO). *Tα1-Cre Ikbkap* CKO mice display many hallmarks of FD symptoms such as small stature, kyphosis, and gait disturbances and typically die by 6 months because of progressive neurodegeneration (Chaverra et al., unpublished observations). In the retina, *Tα1-Cre* is almost exclusively expressed in the RGCs, and deletion of *Ikbkap* in RGCs initially causes slow, progressive loss of RGCs and inflammation in the optic nerve. Interestingly, the RGC loss is subtype specific, with melanopsin^+^ RGCs being resistant to degeneration. In addition, the RGC loss shows regional bias, initiating from the temporal retina followed by spreading throughout the retina. The loss of RGCs is subsequently followed by progressive photoreceptor degeneration, glial activation, and disruption of retinal layers. Because the *Ikbkap* gene is broadly expressed throughout the nervous system in both mice and humans (Mezey et al., 2003), and our *Ta1-cre* was active in many neuronal populations outside of the retina in both the central and peripheral nervous system (Chaverra et al., unpublished observations), we cannot conclude whether the RGC deficits observed in both FD patients and this mouse model were due to direct or indirect consequences of *Ikbkap* loss in the retina.

FD is characterized by both developmental and progressive adult neuropathies. Several mouse models of FD have demonstrated that *Ikbkap* is required for PNS development (Dietrich et al., 2011; Hunnicutt et al., 2012; George et al., 2013; Abashidze et al., 2014; Jackson et al., 2014). We have previously shown, in another CKO FD mouse model in which deletion of *Ikbkap* was restricted to the neural crest lineage, that sensory and sympathetic neurons died by caspase 3– and p53-mediated apoptosis during development (George et al., 2013). In the present study, we demonstrated that *Ikbkap* is expressed in retinal neurons in the GCL as early as E15.5 (Fig. 1*A*). In *Tα1-Cre Ikbkap* CKO retinas, Cre expression was detected in cells in the GCL by E17.5 (Fig. 2*A–C*); however, we did not observe an abnormal number or morphology of RGCs in the mature retina at 1 month (Fig. 5). These results suggest that *Ikbkap* is not required for RGC development.

Visual impairment in FD patients frequently begins at an early age and can progress to legal blindness by their thirties. Until recently, the blindness was thought to be a consequence of inadequate sympathetic and sensory innervation of the eye, since a hallmark of the disease is reduced function of the sensory and autonomic nervous systems (Goldberg et al., 1968; Liebman, 1968; Gold-von Simson and Axelrod, 2006). Although mild to moderate corneal opacities are observed in most eyes of FD patients, none of the eyes had opacities too dense to prevent retinal examinations through the cornea (Mendoza-Santiesteban et al., 2014). Many older CKO eyes displayed mild corneal opacities as well (data not shown). Strikingly, recent clinical studies have revealed that FD patients show decreased visual acuity, poor color vision and central visual field loss, temporal optic nerve pallor, and delay in visual evoked potentials due to loss of RGCs (Mendoza-Santiesteban et al., 2012, 2014). Optical coherence tomography analysis of FD patients showed that their retinas displayed loss of the retinal nerve fiber layer, a sign of RGC loss, predominantly in the temporal retina, and the loss was greatest in older FD patients (Mendoza-Santiesteban et al., 2014). CKO retinas showed phenotypes identical to those of FD patients: progressive RGC loss that affects the temporal retina more severely than the nasal retina (Fig. 5). We also observed optic nerve inflammation (Fig. 6), photoreceptor degeneration (Fig. 8), and disruption of retinal layers (Fig. 3). Whether FD patients also experience these phenotypes has not been reported to date. Understanding the complete course of retinal degeneration, including retinal cell types affected in the absence of *Ikbkap*, is essential in the selection of therapeutic targets to prevent blindness in FD.

Two other retinal disorders, Leber hereditary optic neuropathy (LOHN) and dominant optic atrophy (DOA), share remarkably similar retinal phenotype and disease progression with FD patients and our CKO mice. LOHN and DOA are also characterized by loss of vision due to slow, progressive RGC degeneration, with the more metabolically active temporal RGCs being the first to be affected (Newman and Biousse, 2004; Chevrollier et al., 2008; Zanna et al., 2008; Carelli et al., 2009; Kirches, 2011; Yu-Wai-Man et al., 2014). Interestingly, both of these RGC disorders are considered mitochondrial diseases: the genes that are mutated function in mitochondria. Yu et al. (2012) developed a LOHN model in which the mice carried a mutation in NADH dehydrogenase subunit 4 in the retina. Retinal phenotypes of 1-year-old LOHN retinas were remarkably similar to those of FD retinas in the present study: all mutant animals display RGC degeneration, and some also show disruption of retinal layers as well as loss of neurons other than RGCs (Yu et al., 2012). This similarity in phenotype may indicate that the demise of retinal neurons in FD may also result from pathological processes shared with LOHN and DOA—perturbations in mitochondrial function. In support of this hypothesis, a recent study in yeast shows that wobble uridine modification by the Elongator complex, which includes IKAP/ELP1, is essential for mitochondrial function under stress (Tigano et al., 2015), further suggesting perturbations in mitochondrial function in FD neurons. Future studies in our FD retinal models will also be investigating mitochondrial function.

A growing body of literature shows that ipRGCs are resistant to injury or degeneration in many diseases compared with conventional RGCs (La Morgia et al., 2011; Feigl and Zele, 2014; Cui et al., 2015). ipRGCs are shown to survive after optic nerve injury or transection in rats and mice (Robinson and Madison, 2004; Li et al., 2008; Perez de Sevilla Muller et al., 2014), although the mechanism of cell survival is unknown. ipRGCs are also protected from *N*-methyl-d-aspartate–induced excitotoxicity in mice (DeParis et al., 2012). In patients with mitochondrial neuropathies, including LOHN and DOA, ipRGCs survive even in the presence of extensive loss of conventional RGCs (La Morgia et al., 2010). The pupillary light reflex, which requires functional ipRGCs (Chen et al., 2011), is also spared in these patients (Moura et al., 2013). Again, the mechanisms for ipRGC survival in these mitochondrial neuropathies are not understood. Several hypotheses have been presented, including a neuroprotective role for melanopsin itself, neuroprotection via high levels of pituitary adenylate cyclase-activating polypeptide expressed in ipRGCs, or unique properties of mitochondrial metabolism in ipRGCs (La Morgia et al., 2011; Feigl and Zele, 2014). However, ipRGCs and their function are not spared in all neurodegenerative diseases: ipRGC function is altered in patients with age-related macular degeneration (Cui et al., 2015; Maynard et al., 2015), in patients and animal models with glaucoma (Cooper and Mure, 2008; Drouyer et al., 2008; Wang et al., 2008; Perez-Rico et al., 2010; Feigl et al., 2011; Kankipati et al., 2011; Nissen et al., 2014), and in Alzheimer’s disease (La Morgia et al., 2016). Resistance of melanopsin^+^ ipRGCs in our *Ikbkap* CKO retinas to degeneration (Fig. 7*D*, *E*) and similarities in overall retinal phenotype with other mitochondrial retinopathies (e.g., LOHN and DOA) potentially point to a shared disruption in mitochondrial function. It is worth mentioning, however, that although the GFP Cre reporter was expressed in ipRGCs, it is theoretically possible that the resistance to death of ipRGCs in our CKO is due to residual IKAP protein expression.

Although we have shown that loss of *Ikbkap* in CKO mice causes slow, progressive RGC degeneration (Fig. 5) followed by photoreceptor degeneration (Fig. 8) and retinal disorganization (Fig. 3), these phenotypes were observed in *Tα1-Cre Ikbkap* CKO mice that have a systematic neuronal deletion of *Ikbkap* causing a severe progressive neuropathy that could indirectly generate these retinal deficits. In addition, with this model we are unable to analyze the consequence of *Ikbkap* loss in retinal cell types other than RGCs, since expression of *Tα1-Cre* is primarily restricted to RGCs in the retina (Fig. 2). Therefore, we are currently generating retina-specific *Ikbkap* CKO mice to analyze direct consequences of *Ikbkap* loss in all retinal neuron subtypes and be able to distinguish autonomous versus nonautonomous requirements for *Ikbkap* in various retinal populations. Nonetheless, this is the first study that not only identified the endogenous expression pattern of *Ikbkap* in the retina, but also characterized the retinal phenotype and pathology that are the consequences of the absence of *Ikbkap* in RGCs. The phenotypes observed in our CKO retinas were remarkably similar to clinical presentation of FD patients’ retinas (Mendoza-Santiesteban et al., 2012, 2014).

Although one of the most debilitating hallmarks of FD as patients age is progressive blindness, the pathophysiological mechanisms underlying the loss of vision have not been the focus of any study. With our *Tα1-Cre Ikbkap* CKO model of FD blindness, not only are we able to investigate the course and causes of disease progression, but we also have established an *in vivo* model system for testing potential therapeutics for mitigating the progressive optic neuropathy in FD patients.

## Synthesis

Reviewing Editor: Siegrid Loewel, University of Goettingen Decisions are customarily a result of the Reviewing Editor and the peer reviewers coming together and discussing their recommendations until a consensus is reached. When revisions are invited, a fact-based synthesis statement explaining their decision and outlining what is needed to prepare a revision will be listed below. The following reviewers agreed to reveal their identity: Michel Cayouette, Donald Zack

This is a revised paper that was originally submitted to J. Neurosci. The authors have improved the manuscript according to the previous reviewers' comments, or have presented reasonable arguments for why it was not possible to address certain points. Both reviewers agree in that important results on a new mouse model for retinal changes in FD are presented.

I ask the authors to consider the following minor changes to the present version of the manuscript:

You note that ipRGCs are resistant to degeneration caused by the absence of IKBKAP. This is a potentially important finding and seems probably correct. However, in follow-up of this issue that was mentioned in the prior reviews, it would be good if the authors would include in the discussion that it is not totally clear that IKBKAP is expressed in ipRGCs and that its expression is totally deleted in the conditional KO animals (the indicated GFP expression in the reporter mice is not sufficient to prove the point).

The authors suggest that the late PR degeneration is probably secondary since the conditional reporter mice do not show significant Cre expression in PRs (Figure 2). However, the PR Cre data is for 1 month old mice and the PR degeneration takes place many months later. There seems to be no demonstration that Cre expression is not turned on in PRs with aging, so it is possible that such late expression in PRs could explain their late degeneration. This possibility should be mentioned in the discussion.
